# Biodiversity as a multidimensional construct: a review, framework and case study of herbivory's impact on plant biodiversity

**DOI:** 10.1098/rspb.2015.3005

**Published:** 2016-12-14

**Authors:** S. Naeem, Case Prager, Brian Weeks, Alex Varga, Dan F. B. Flynn, Kevin Griffin, Robert Muscarella, Matthew Palmer, Stephen Wood, William Schuster

**Affiliations:** 1 Department of Ecology, Evolution and Environmental Biology, Columbia University, 1200 Amsterdam Avenue MC5557, New York, NY 10027, USA; 2 Earth Institute Center for Environmental Studies, Columbia University, New York, NY 10027, USA; 3 The Arnold Arboretum of Harvard University, Boston, MA 02130, USA; 4 Department of Ecoinformatics and Biodiversity, Aarhus University, 8000 Aarhus C, Denmark; 5 Yale School of Forestry and Environmental Studies, New Haven, CT 06511, USA; 6 Black Rock Forest Consortium, Cornwall, NY 12518, USA

**Keywords:** biodiversity, ecosystem function, ecosystem services, multiple dimensions of biodiversity, conceptual framework

## Abstract

Biodiversity is inherently multidimensional, encompassing taxonomic, functional, phylogenetic, genetic, landscape and many other elements of variability of life on the Earth. However, this fundamental principle of multidimensionality is rarely applied in research aimed at understanding biodiversity's value to ecosystem functions and the services they provide. This oversight means that our current understanding of the ecological and environmental consequences of biodiversity loss is limited primarily to what unidimensional studies have revealed. To address this issue, we review the literature, develop a conceptual framework for multidimensional biodiversity research based on this review and provide a case study to explore the framework. Our case study specifically examines how herbivory by whitetail deer (*Odocoileus virginianus*) alters the multidimensional influence of biodiversity on understory plant cover at Black Rock Forest, New York. Using three biodiversity dimensions (taxonomic, functional and phylogenetic diversity) to explore our framework, we found that herbivory alters biodiversity's multidimensional influence on plant cover; an effect not observable through a unidimensional approach. Although our review, framework and case study illustrate the advantages of multidimensional over unidimensional approaches, they also illustrate the statistical and empirical challenges such work entails. Meeting these challenges, however, where data and resources permit, will be important if we are to better understand and manage the consequences we face as biodiversity continues to decline in the foreseeable future.

## 1. Introduction

*Biodiversity* defies easy definition, but we value it nonetheless, much the way we value *justice*, *freedom* and *nature*, similarly difficult terms to define. For research and policy concerning biodiversity, however, concrete definitions of biodiversity are important and while definitions of biodiversity vary enormously (e.g. [1–4]), one common feature of these varied definitions is that biodiversity is inherently multidimensional. That is, *biodiversity* refers to multiple elements of variability of life on the Earth, be it taxonomic, functional, phylogenetic, genetic, trophic or other ways that life's forms and functions vary [5–8]. Thus, biodiversity has its lowest non-zero value when an ecosystem contains a single recently evolved species, consisting of one genetically homogeneous population that is small in its geographical extent and narrow in its range of habitats. Biodiversity has its highest value when there are many species that represent a broad taxonomic range, with some species recently evolved, others ancient and all made up of many genetically heterogeneous populations that exhibit interactions within and among other populations across the landscape through emigration and immigration and describe a complex interaction network with many nodes and many species per node. Thus, biodiversity is not defined by a unidimensional continuum or spectrum, but a multivariate, hyperdimensional space in which each dimension represents one element of life's diversity. Measures of biodiversity would include the community’s location in that hyperdimensional space, the volume that replicate communities occupy, and the distribution and density of points (each point being one community) in that space. Such a definition of diversity is difficult to employ in research and policy, but it more accurately reflects what biodiversity means.

Multidimensional biodiversity research, or research in which two or more dimensions of biodiversity are simultaneously investigated, is currently a surprisingly small field in spite of widespread recognition of its importance. These studies generally explore how insights gained from traditional unidimensional biodiversity research differ from those derived when multidimensional approaches are taken (e.g. [7–14]). For example, Muscarella *et al*. [12] found that functional diversity (FD) declined during tropical forest succession while phylogenetic diversity (PD) increased, illustrating that different dimensions of diversity may differ in temporal trends and illuminating the role of physiology and history in governing succession.

Objectives of multidimensional studies that do not include intrinsic or extrinsic variables are often comparative studies. For example, Stevens & Gavilanez [7] examined the structure of species, or taxonomic diversity (TD), PD, FD and morphological diversity (e.g. phenetic, which might be considered related to FD) in bat communities, comparing natural communities to those constructed from random draws (i.e. null communities) of species from the regional pool. They found that natural bat communities exhibit higher degrees of dimensionality than null communities, which points to a hitherto unappreciated structure in biodiversity. In another example, Strecker *et al*. [15] used TD, FD and PD in their prioritization of conservation areas for freshwater fish communities rather than simply basing such priorities on TD alone. Similarly, in their study of bird diversity in protected areas of France, Devictor *et al*. [16] identified instances of congruence and mismatch among TD, FD and PD. Given potential mismatches, where some sites may have high values for one measure and low values for others, the authors propose using metrics that integrate across multiple dimensions of biodiversity and several studies have explored multidimensional metrics of biodiversity (e.g. [5,17,18]).

Multidimensional studies can provide greater insight into the mechanisms underpinning biodiversity's influence on ecosystem properties than unidimensional studies. For example, both Lasky *et al*. [19] and Muscarella *et al*. [12] found that correlations between TD, FD, and PD and stand biomass (i.e. an ecosystem property) were not constant and varied during forest succession, pointing to different community dynamics among trees over time. Another example is Cadotte *et al*. [6], which analysed the degree to which ecosystem productivity can be explained by different dimensions of biodiversity. They found that primary productivity (i.e. the ecosystem property) was better captured by PD than FD. In a meta-analysis, Flynn *et al*. [14], using a variety of methods, including structural equation modelling (SEM), found that phylogenetic diversity and FD both explain more variation in biodiversity—ecosystem–functioning relationships than species richness, but with distinct, uncorrelated mechanisms as drivers.

Recent biodiversity studies point to the importance of a multidimensional perspective in observational, comparative and experimental biodiversity research; however, few studies employ a truly multidimensional approach. Here, we examine trends in the current literature to explore the uptake of multidimensional approaches by the research community and develop a conceptual framework based on our review of the literature. By way of illustration, we provide a case study that employs this framework to examine how multidimensional biodiversity influences on understory plant cover (an ecosystem property that serves as a proxy for production) at Black Rock Forest, New York, are impacted by herbivory. Our case study serves to illustrate the framework and contrasts univariate, multivariate and multidimensional approaches to understanding the relationship between biodiversity and ecosystem properties. We discuss the implications of our findings from our review, framework and case study on the value and challenges of moving biodiversity research forward towards a multidimensional approach in this age of mass extinction.

## 2. Material and methods

### (a) Literature review

Using the BIOSIS database, selecting for abstracts in the English language, we searched for the terms ‘dimensions of biodiversity’, ‘taxonomic diversity’, ‘species richness’, ‘phylogenetic diversity’, ‘functional diversity’, ‘trait diversity’ and ‘functional trait’. The term ‘dimensions of biodiversity’ first appeared in the BIOSIS database in 1997. From that year we tallied the number of studies each year for these seven terms, removing articles from non-environmental journals. The counts were then normalized by dividing by the total number of results for the search term ‘biodiversity’ for each year. Beginning in the year 1997, we further reviewed abstracts to determine which dimension or dimensions of biodiversity were used (TD, PD, FD or other). For 1997–2007, we surveyed the first 20 relevant papers found. For the last four years (2008–2012), we surveyed 50 papers per year. Abstract results in BIOSIS were randomized to avoid potential biases based on number of citations, journal popularity and publishing date. In our selection criteria, we limited the review to articles where the authors used biodiversity as a variable to answer an ecological question, either researching the impact of biodiversity on other factors, or how different factors, biotic or abiotic, impact biodiversity. Thus, this analysis does not include papers that strictly sought to describe or quantify biodiversity.

### (b) Conceptual framework

We developed a conceptual framework based on current approaches in multidimensional biodiversity research that can facilitate its expansion. Although changes in biodiversity are driven by many factors, contemporary research devotes considerable effort to the study of anthropogenic divers [8,20–28]. We, therefore, emphasize anthropic drivers, but the distinction between natural and anthropic drivers is not important to our framework.

Our framework includes metrics that quantify the multiple dimensions of biodiversity. Biodiversity is quantified in many ways, often using indices for different dimensions of diversity. There are many indices of TD, FD, PD and other dimensions of biodiversity [11,29–32], thus in each study, each dimension is likely to have multiple metrics that quantify patterns of richness and dispersion of taxa along that dimension. For this reason, we consider all dimensions to covary with taxonomic richness, which is most likely to be species, but as molecular tools evolve, what constitutes a taxonomic unit is evolving, especially for prokaryotes.

In our conceptual framework, we elected to consider the influence of the number of taxa as one of the dimensions of biodiversity. In our empirical example to illustrate the framework, the model parallels the framework. The rationale underlying our SEM model is that the number of taxa covaries with most metrics of diversity, but alternative approaches can be taken. We explore three alternative models, using our empirical example, that each incorporate the number of taxa in different ways (see the electronic supplementary material).

Note that in the literature, species richness, or other tabulations of taxa, is frequently considered a measure of TD, thus our treatment of TD in the literature review is different from our treatment in the conceptual framework.

We used the conventions employed in SEM (e.g. [33–36]) for assembling our framework, an approach used in other studies of biodiversity (e.g. [37,38]). This approach focuses on the multiple influences of variables on one another either through direct or indirect linkages or as covariates. These models distinguish between those variables observed and measured (manifest variables) and those that are not measured (latent variables). For example, health, as a variable, cannot be measured directly, but can be treated as a latent variable that, like an indicator, reflects many physiological functions that can be measured (manifest variables). We considered each dimension of biodiversity to be rarely observed, but quantified by a variety of methods, thus dimensions of biodiversity are considered latent variables. Similarly, ecosystem properties were treated as latent variables as they are rarely directly observed, but are measured using proxy variables. System productivity, for example, may be measured as net primary productivity, using a normalized difference vegetation index, as annual leaf litter fall in deciduous forests, or as per cent plant cover in grassland plots.

The framework is deliberately broad in order to accommodate the many drivers or factors recognized to be important in biodiversity effects, but it does not specifically address mechanisms underlying ecosystem response. Mechanisms, such as selection and complementarity, are impacted by changes in community driven by changes in biotic factors, such as predation or herbivory, or abiotic factors, such as climate or pH. Likewise, this framework accommodates the wide array of diversity metrics, but metric selection is likely to influence outcomes. For example, FD is quantified by many metrics, each with different mathematical formulations (e.g. weighted or unweighted by abundance) and different emphases (e.g. emphasizing richness, divergence or both) [29]. While SEM allows for covariance among metrics and sensitivity analyses can be used to compare the relative influences of different metrics on outcomes, it does not provide insight into how or why different metrics have different effects. Our case study illustrates these issues.

### (c) Illustration of the framework: a case study of the multiple dimensions of plant diversity, plant cover and herbivory

To illustrate the framework, we applied our framework to test the hypothesis that herbivory by whitetail deer (*Odocoileus virginianus*), as the driver of biodiversity change, altered biodiversity's influence on plant per cent cover as an ecosystem property. Our approach was to apply the framework to vegetation protected from herbivory and vegetation exposed to herbivory. Deer herbivory impact on vegetation is well studied [39–41], in part, because of concern over excessive densities of deer partly attributable to extirpation of apex predators and unsuccessful management [42]. Thus, we consider excessive deer herbivory to be an anthropogenic driver of current changes in biodiversity. Our illustration considers only three dimensions of biodiversity and a single ecosystem property, but serves to contrast univariate, multivariate, unidimensional and multidimensional approaches in the study of biodiversity's relationship to ecosystem properties.

#### (i) Study system

All plant cover data were collected in 2010 at Black Rock Forest, a 1550 ha preserve located in Cornwall, NY, USA, in the Hudson Highlands Region of southeastern New York State. Black Rock Forest is a mixed hardwood forest, dominated by oak species—with a canopy composition of 67% oak and 33% non-oak [43]. The study plots are located on the north slope of Black Rock Mountain (41.45° N, 74.01° W) within a long-term oak removal experiment comprising four treatments replicated in three blocks: 100% oaks girdled, 50% oaks girdled, 100% non-oaks girdled and a control. For this study, we evaluated plant diversity inside enclosures (protection from deer herbivory) and outside of enclosures (susceptible to herbivory), and we did not consider oak removal treatments. Oak removal was considered a treatment that uniformly enhanced stand variability across the 12 plots within which deer exclosures were located. The plots (75 × 75 m) and their respective canopy treatments were established in 2009. Each plot contains ten fenced and unfenced areas, each 5 × 10 m, arranged in a grid in a central 25 m × 25 m area to avoid the plot edge.

#### (ii) Forest understory survey

There were 93 taxa, 13 of which could not be readily determined during surveys. Of these 80 determined species, only 31 had trait data necessary to estimate FD, thus we restricted all analyses to these. These species, however, were the most abundant and accounted for more than 85% of the understory plant cover. Plant cover data were collected in August 2010 from 240 1 m^2^ vegetation plots distributed across elevation and canopy disturbance gradients. The per cent cover of each species of vascular plant present in the ground layer vegetation was estimated visually for each plot. This includes estimates of all cover for all plant material within 2 m of the ground; vegetation > 2 m tall, including the overhanging canopy, was not considered part of the ground layer vegetation and was, therefore, not estimated. Per cent cover is estimated to the nearest 1%, though species with ≪1% cover were recorded as 0.1%. Certain taxa were not readily identifiable to species during the sampling (primarily small seedlings and non-reproductive grasses and sedges) and those are excluded from these analyses. Nomenclature follows the USDA PLANTS database (plants.usda.gov). To avoid pseudoreplication, plots were combined and means taken within blocks, reducing the total number of replicate plant cover surveys to 24.

#### (iii) Trait data

We selected three plant functional traits that are fundamental measures of plant physiology and productivity that yielded trait data for the greatest number of species present in our community. These three traits were: (i) specific leaf area (SLA), (ii) leaf nitrogen content (LNC) and (iii) leaf phosphorus content (LPC). All three traits are relevant aspects of the ‘leaf economic spectrum’ by which species exhibit trade-offs between high rates of photosynthesis and leaf tissue longevity [44–46]. All trait data were obtained from the TRY plant trait database [47]. For each species, three individual trait measurements, obtained from the TRY database, were averaged and used as mean trait values for each species in our FD analyses.

### (d) Diversity metrics

All diversity indices and total cover were calculated using the 31 species present in the community for which functional trait measurements could be obtained from the TRY Plant Trait Database [47]. For abundance-weighted indices, species abundances were considered to be the per cent cover of each species within a plot. FD was calculated using the standardized values of three functional traits: SLA, LNC and LPC. To characterize FD, we used abundance-weighted measures of functional evenness and functional divergence [48], implemented in the R Package ‘FD’ [49,50]. Functional evenness represents the regularity with which species are distributed throughout a multidimensional functional space. If the species in a community are clustered within functional space, functional evenness will be low and vice versa. Similarly, functional divergence describes the distribution of species throughout a multidimensional space, but captures the degree to which species are close to the centre of the functional space or close to the edges of the functional space. Those communities with a high proportion of species clustered near the centre of functional space will have low values of functional divergence, while communities with high proportions of species near the edges of the space will have high functional divergence values. These two indices are designed to be independent of species richness, and the equations used to calculate functional evenness and functional divergence can be found in [48]. A third metric of FD proposed by Villéger *et al*. [48] is functional richness, which is calculated based on the total volume of a multidimensional space defined by the functional traits of the species in a community. This metric, unlike functional evenness and functional divergence, is not independent of species richness [48]. In order to avoid issues of multicollinearity across FD, PD and TD, we did not include functional richness or species richness, which are highly correlated with Faith's PD (see electronic supplementary material, table S2, which provides pairwise correlations among all biodiversity metrics).

The PD was estimated using two indices: abundance-weighted mean pairwise distance (MPD) and Faith's PD [51]. These metrics were calculated using the Phylocom software [51], with phylogenetic relationships and branch lengths obtained from the angiosperm supertree [52]. MPD captures the mean sum of the phylogenetic branch lengths separating species pairs within a community, so it is not highly sensitive to species richness, and has been shown to be an effective predictor of ecosystem function [53]. Faith's PD represents the sum of all branch lengths connecting the members of a community [54], and is highly correlated with species richness. As such, Faith's PD was included to provide a general index for the total extent of phylogenetic, taxonomic (i.e. species richness) and functional (i.e. multidimensional functional space) diversity within a community.

The TD was measured using the Simpson and Shannon indices of diversity, calculated using Phylocom [51]. These estimates of TD are frequently used in ecological studies to represent the evenness of species abundances, and the extent to which a community is dominated by a limited number of species.

### (e) Analyses

#### (i) Influence of herbivory on multiple dimensions of biodiversity

We employed two analytical approaches in our case study to contrast unidimensional with multidimensional approaches. First, we employed conventional univariate and multivariate methods employed in unidimensional biodiversity research. Second, we employed the SEM framework described above.

*Unidimensional*, *univariate and multivariate statistical approaches.* We used analyses of variance (ANOVAs) models to examine how protection from herbivory impacted per cent cover, species richness and all metrics of biodiversity independently, including number of taxa as a metric. We also ran multivariate analyses of variance (MANOVAs) to examine the relationships between protection from herbivory and all biodiversity metrics.

*SEM—framework approach*. We used SEM to analyse the relationship between different metrics of biodiversity (observed variables), dimensions of biodiversity (unobserved or latent variables) and the ecosystem function or service, total understory cover. SEM is a complex set of statistical techniques that is reviewed in several studies (e.g. [33–35,55]), thus we address just a few issues specific to its use in multidimensional biodiversity research.

In SEM, there are a number of means for estimating model fit to the data, each of which have pros and cons [56–61], but it is important to note that rejection of fit does not mean that the model is incorrect, only that the data are not well described by the model. When data are not well fit by the model, inference necessarily requires either additional data or an alternative model.

We used AMOS [56] to conduct all SEM analyses and SYSTAT [62] for ANOVAs and MANOVAs.

## 3. Results

### (a) Literature survey

**Figure 1.**
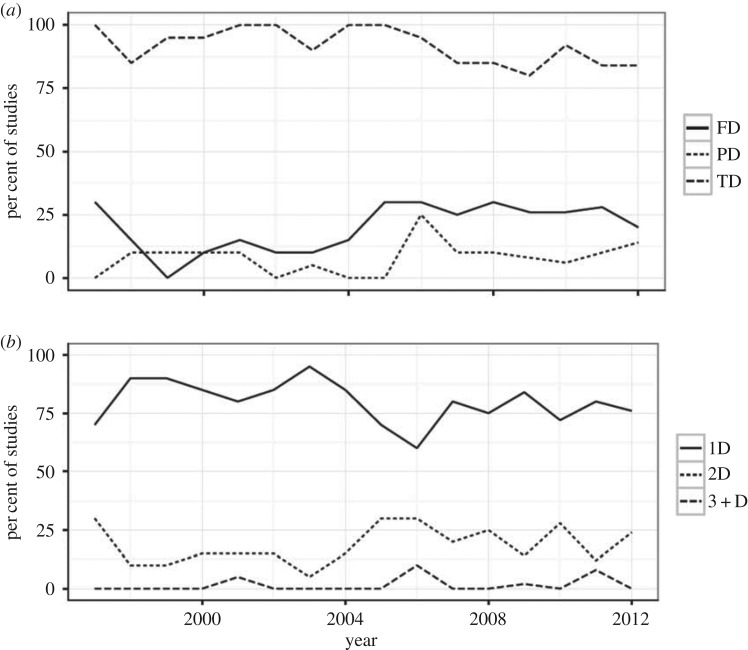
Temporal trends in the literature for multiple dimensions of biodiversity. These figures illustrate little in the way of consistent trends in the number or type of biodiversity dimension used in ecological research. (*a*) Per cent of sampled biodiversity studies that measured either taxonomic, functional or phylogenetic diversity (TD, FD or PD, respectively). In 2001, for example, 95% of the sampled studies included measures of TD, 15% measured functional diversity and possibly other metrics, while 5% measured phylogenetic diversity and possibly other metrics. (*b*) Another way to look at these data is the number of dimensions in a study. In 2001, for example, 5%, 15% and 80% of all studies sampled included 3 or more (3 + D), two (2D) and one (1D) dimensions, respectively. Note that many studies in this literature survey consider number of taxa, such as species richness, to be a measure of TD, but our framework considers taxonomic richness to be a covariate of TD and other dimensions of biodiversity.

Our quantitative literature survey showed no apparent or weak trends (no significant correlations with time for any measure, either Pearson's or Spearman's-rank coefficient, and no significant auto- or partial autocorrelations with the exception of FD, indicating a positive correlation at a time lag of 1 year and for three dimensions at a time lag of 5 years, the latter owing to the sparsity of studies with a few occurrences in 2001, 2006 and 2011). Studies of biodiversity show a strong, steady dominance by analyses that use TD, a smaller number of studies using FD and fewer using PD (figure 1*a*). In tallying the number of dimensions of biodiversity, research is dominated by unidimensional studies (70–80% of studies; figure 1*b*). While there is a rise in the number of studies exploring two dimensions, peaking at 30% of studies surveyed, the number of studies using three or more dimensions is extremely low (less than 10%).

### (b) A multidimensional biodiversity framework

**Figure 2.**
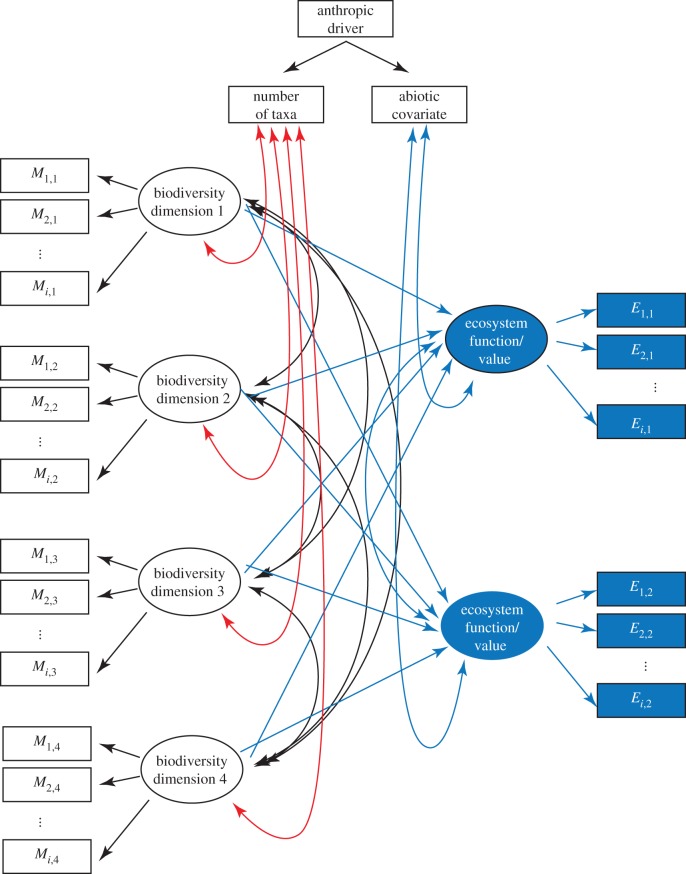
A conceptual framework for the structural relationship between multiple dimensions of biodiversity, ecosystem functions and their values, covariates with biotic richness and the abiotic environment, and anthropic drivers. Following conventions used in structural equation modelling, dimensions of biodiversity (taxonomic, functional, phylogenetic, etc.) are not observed or measured, but are latent variables assessed by different metrics, here labelled as *M_i_*_,*j*_ for the *i*th metric for the *j*th dimension. Note that number of taxa is considered a covariate of diversity dimensions and not a dimension itself. Ecosystem functions are similarly rarely observed, but measured in a variety of ways. Soil fertility, for example, will be a function of microbial diversity, soil organic matter, soil moisture and nutrient availability. Stability may be a measure of the ratios of function metrics pre- and post-perturbation, such as the ratio of the sum of species-specific plant production prior to a drought and the summed production after a drought. Abiotic factors, such as temperature, precipitation, insolation, N deposition and other physical/chemical factors will covary with ecosystem functions and their values. Biodiversity dimensions will covary with taxonomic richness in the sense that most metrics of diversity increase with richness. Finally, anthropic drivers, such as the extirpation, overexploitation, or restoration and conservation of species, will directly influence the number and will influence abiotic factors, such as the impacts of anthropogenic greenhouse gas warming and changes in local and regional temperature and precipitation. Colours are arbitrarily assigned and are simply for clarity. Black arrows, ovals and rectangles represent paths, latent variables and observed (measured or manifest) variables, respectively. Blue-coloured elements represent ecosystem functions or services. Red-coloured elements represent covariation between all dimensions of biodiversity and number of taxa, treated here as an exogenous variable. A single anthropic driver of biodiversity change (e.g. climate change, apex predator extirpation, land degradation) is shown at the top.

Based on our reading of current multidimensional biodiversity research, the conceptual framework we propose is presented in figure 2. Our framework took the simplifying steps of

(1) using only four dimensions of biodiversity (with three metrics per dimension shown),
(2) using only two ecosystem functions (with three metrics shown),
(3) using only one abiotic driver,
(4) using only one anthropic driver and
(5) focusing on covariance between metrics and number of taxa but not among metrics.

Despite these four simplifying effects, 41 paths are present (figure 2).

### (c) Influence of herbivory on multiple dimensions of biodiversity

*Unidimensional*, *univariate and multivariate statistical approaches.* Protection from deer herbivory did not significantly alter total cover (ANOVA; d.f. = 1, 22; *F* = 1.11, *p* = 0.30) or taxonomic richness (ANOVA; d.f. = 1, 22; *F* = 0.17, *p* = 0.69) of total cover. Although neither taxonomic richness nor total cover was affected by protection from deer herbivory, multivariate analyses of the responses of biodiversity metrics to the treatment revealed significant effects. The vector of biodiversity indices showed a significant response to protection from deer herbivory (MANOVA; Wilk's Lambda *p* < 0.001; Pillai Trace *p* < 0.01; Hotelling-Lawley Trace *p* < 0.001; Roy's greatest Root *p* < 0.001). The standardized canonical coefficients suggest that the order of dependent variable contribution to overall MANOVA results was Simpson's, Shannon, FD_divergence_, FD_evenness_, MPD and Faith PD, in order of magnitude. MPD and Shannon had opposite effects (sign of coefficient negative) to the others. We note that all univariate tests revealed significant responses of the different biodiversity metrics to protection from herbivory (ANOVA, *p* < 0.001).

Multiple linear regressions showed marked differences between protected and unprotected plots. For the unprotected plots, stepwise deletion removed all but one metric of biodiversity as independent variables, yielding a model that contained only number of taxa as the independent variable and showed a positive association between diversity and total cover (coefficient = 3.64, s.e. = 0.88, *R*^2^ = 0.59, *p* < 0.01). By contrast, when protected from herbivory, no significant association was detected by multiple linear regression (*R*^2^ = 0.05, *p* = 0.44) and stepwise deletion did not yield a significantly better model fit.

**Figure 3.**
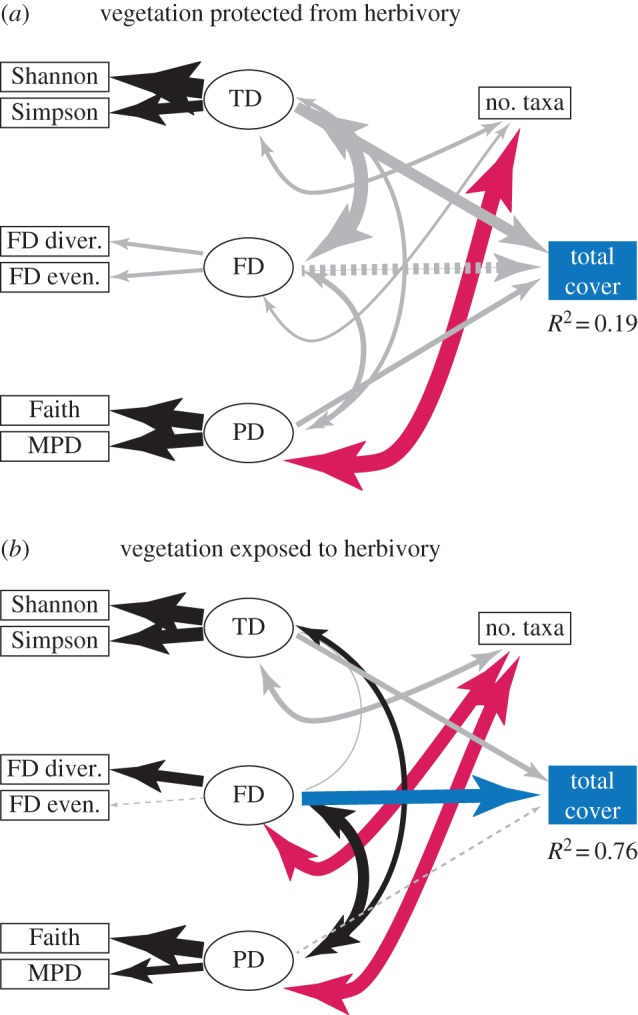
(*a*,*b*) Application of the conceptual framework applied to the response of vegetation biodiversity to deer herbivory. Table 1 provides the coefficients and significance values used to prepare the figures. Width of arrows (paths) represents magnitude of coefficient. Double-headed paths are correlations, while single-headed are paths. Grey paths are non-significant (*p* > 0.05). Dashed lines represent paths with negative coefficients. Latent variables are taxonomic diversity (TD), functional diversity (FD) and phylogenetic diversity (PD). TD is calculated using two indices, the Shannon diversity index and the Simpson index; FD is calculated from functional evenness and functional divergence; and PD is composed of the abundance-weighted MPD and Faith's PD. ‘No. taxa’ represents number of species in this application of the framework. *R*^2^ is the squared multiple correlation that reflects proportion variance explained by the SEM model in ‘total cover’, the selected ecosystem property in this study. Note that number of taxa is considered a covariate of TD, not a metric of TD, thus the Simpson and Shannon metrics are treated as distinct from, but influenced by species richness. Alternative approaches are presented in the electronic supplementary material. (Online version in colour.)

*SEM—framework approach*. Employing the framework presented in figure 2, SEM revealed considerable change in the influence of different dimensions of biodiversity on total cover when protected from deer herbivory (figure 3). Neither TD nor FD had significant influence on cover, with PD showing the weakest positive influence. The influence of multiple dimensions of biodiversity on total cover, however, was weak (only 19% of variability in total cover explained by the three dimensions of biodiversity) and the model fit poor (root mean square error of approximation (RMSEA) = 0.55, *p* < 0.001), thus these results are presented only for illustrating the framework.

**Table 1.** Structural equation modelling (SEM) coefficients, standard errors (s.e.) and probabilities (*p*) for understory vegetation response to protection from or exposure to deer herbivory at Black Rock Forest. One-way arrows represent paths between variables, while two-way arrows represent covariance. Standardized coefficients for covariances are correlation coefficients. Critical ratios (C.R.) are the estimated coefficient divided by its standard error. *p*-Values lower than 0.001 are presented as ‘<0.001’. We used the standard critical *α* of *p* < 0.05 to determine which paths were significant, as shown in figure 3*a*,*b*.

	estimate (s.e.)	standardized	C.R.	*p*-value
vegetation protected from herbivory				
Shannon ← TD	0.59 (0.11)	1.28	5.34	<0.001
Simpson ← TD	0.14 (0.50)	0.75	2.68	0.007
FD_Divergence ← FD	0.03 (0.03)	0.27	1.11	0.267
FD_Evenness ← FD	0.05 (0.05)	0.27	1.10	0.270
Faith ← PD	0.14 (0.03)	0.96	4.33	<0.001
MPD ← PD	0.16 (0.04)	0.87	3.64	<0.001
Total_Cover ← TD	11.97 (19.16)	0.68	0.63	0.532
Total_Cover ← FD	−11.31 (16.77)	−0.64	−0.68	0.500
Total_Cover ← PD	7.04 (10.20)	0.40	0.69	0.490
TD ↔ FD	0.93 (0.52)	0.93	1.78	0.075
PD ↔ FD	0.35 (0.78)	0.35	0.45	0.652
PD ↔ TD	0.27 (0.18)	0.27	1.48	0.140
nTaxa ↔ TD	0.76 (0.57)	0.29	1.35	0.177
nTaxa ↔ FD	0.65 (2.10)	0.24	0.31	0.756
nTaxa ↔ PD	2.60 (0.57)	1.01	4.73	<0.001
vegetation exposed to herbivory				
Shannon ← TD	0.43 (0.07)	1.20	5.94	<0.001
Simpson ← TD	0.12 (0.04)	0.79	2.98	0.003
FD_Divergence ← FD	0.10 (0.04)	0.73	2.82	0.005
FD_Evenness ← FD	−0.02 (0.04)	−0.11	−0.47	0.638
Faith ← PD	0.18 (0.04)	1.06	5.20	<0.001
MPD ← PD	0.10 (0.05)	0.57	2.04	0.041
Total_Cover ← TD	6.19 (4.61)	0.35	1.34	0.179
Total_Cover ← FD	16.51 (5.41)	0.92	3.05	0.002
Total_Cover ← PD	−2.30 (5.29)	−0.12	−0.44	0.660
TD ↔ FD	−0.03 (0.26)	−0.03	−0.110	0.913
PD ↔ FD	0.71 (0.21)	0.71	3.30	<0.001
PD ↔ TD	0.43 (0.19)	0.43	2.19	0.028
nTaxa ↔ TD	1.64 (0.96)	0.42	1.71	0.087
nTaxa ↔ FD	3.25 (1.10)	0.83	2.95	0.003
nTaxa ↔ PD	3.59 (0.91	0.92	3.97	<0.001

In contrast with vegetation protected from herbivory, in the absence of protection from deer herbivory, FD had the strongest influence, nearly threefold that of TD, with PD making the lowest contribution and opposite in sign (table 1, figure 3). Further, the three dimensions of biodiversity collectively explained 76% of variability in total cover, nearly fourfold that observed in protected plots, though the model fit remained poor (RMSEA = 0.30, *p* < 0.05), thus these results are, as above, presented only to illustrate the framework.

The SEM suggests that differences in the influence of multiple dimensions of biodiversity on total cover may be driven by changes in the number of taxa observed in each plot, even though means were not significantly different.

Note that the ecosystem property of total cover was directly observed as a single metric (figure 3*a*,*b*), which differs from the conceptual framework where an ecosystem property may be a function of several measured variables (figure 2).

## 4. Discussion

Biodiversity is well recognized as a multidimensional construct and several studies have highlighted the differences in outcomes when one employs a multidimensional approach rather than a unidimensional approach. Yet, an analysis of scientific literature over the past 17 years shows that the majority of existing work examines just one dimension of biodiversity, and that very few studies incorporate more than two dimensions, indicating little exploration of the principle that biodiversity is inherently multidimensional either because of author choice or data limitation (figure 2).

Given that biodiversity research is dominated by unidimensional studies, most consisting of studies of TD (including number of taxa as a metric), the accuracy and utility of these findings may be limited in the absence of appreciating the true complexity underlying how and why such influences occur. The implications for conservation, restoration and policy derived from unidimensional biodiversity studies are likely to be similarly limited in their accuracy and utility. Our framework and the worked example of the impacts of white tail deer herbivory on plant diversity and its influence on production, however, show how future multidimensional research can address these shortcomings.

Our conceptual framework shows just how extraordinarily complex even a simple model of the relationship between multiple dimensions of biodiversity, ecosystem functions, the abiotic environment and anthropic drivers can be. Our framework has 42 different paths to be estimated (figure 2), which means that empirical work will be fairly challenging. Rules of thumb for sample size requirement for SEM vary and are unreliable means for insuring power [63], but as a first approximation, they suggest that for 42 paths, a minimum of approximately 420 observations (replicate ecosystems) would be needed per treatment level. We know of no biodiversity study of this magnitude in the current literature. A study of this size is likely to be impractical given current research infrastructure and funding. In our application using Black Rock forest understory vegetation, our model included 18 variables, requiring 180 observations. Our model included only 12 observations; thus, while the model was resolved, the fit was poor using the RMSEA (*p* < 0.0) for both protected and unprotected vegetation, which rejects model fit. There is, however, considerable discussion among researchers concerning how best to estimate model fit, its pros and cons, and the sensitivity of RMSEA to degrees of freedom, size of the variance–covariance matrix and sample size [56–61], thus it is possible that a larger dataset would improve our ability to estimate model fit. As this case study, however, is meant primarily to illustrate an SEM-based approach to analysing multidimensional biodiversity effects, we note that continuing debate over how best to estimate model fit in SEM means caution should be applied in interpreting results. In a similar vein, while standardized coefficients can be greater than unity when replication is low, such results are undesirable and limit interpretation of findings [56]. For this reason, we have not attempted to make specific statements about the influence of protection from herbivory on the relationship between multiple dimensions of biodiversity and total plant cover. Rather, we have reported only that the structure of the relationship between dimensions of biodiversity and ecosystem function (total cover) is qualitatively different between the treatments.

While it is not our intent in this paper to specifically address issues concerning herbivory and vegetation, or the environmental problem created by the anthropic driver of apex species loss, we can make interesting observations about unidimensional versus multidimensional approaches in biodiversity research. We show that when conventional univariate (ANOVAs) or multivariate approaches (MANOVAs and multiple linear regressions) are taken, the outcomes are quite different from what a SEM approach based on our conceptual model reveals. SEM analyses suggest that herbivory may change the relationships among dimensions of biodiversity and our ecosystem function/service of plant cover.

A valuable observation that emerges from our review, framework and case study provided to illustrate the framework, is that biodiversity dimensions are often considered, in the abstract, orthogonal or uncorrelated to one another, but their metrics generally covary. Covariance among biodiversity metrics can be especially problematic when using linear models where collinearity among independent variables must be minimized. An advantage of the SEM framework is that it allows for biodiversity metrics to covary, which obviates the difficulties that can arise if one treats dimensions of biodiversity as orthogonal axes when neither they nor their metrics are. While such covariances were included in our SEM analyses by allowing dimensions to covary with each other and the number of taxa (figure 3; alternatives in electronic supplementary material, figure S1), interpreting ANOVA, MANOVA and multiple regression results is difficult because of the correlations among variables.

A second issue is that biodiversity metrics may employ different approaches to quantifying different dimensions of biodiversity. For example, biodiversity metrics employed in a multidimensional study could consist of a mix of those weighted by abundance and those that are not. In such cases, it becomes unclear if results reflect differences in dimensions or differences in metric formulation used to quantify the different metrics. In our case study, for example, we used only abundance-weighted metrics to minimize possible complications arising from using metrics formulated in significantly different ways, but it remains unclear if other differences in formulation influenced the outcome. In fact, it is possible that some researchers may consider number of taxa and relative abundance as separate dimensions of biodiversity. Future studies could employ sensitivity analyses to see how metric selection influences outcomes.

## 5. Conclusion

Despite widespread recognition of biodiversity being multidimensional, research has been primarily unidimensional in its approach, with simple counts of species being by far the dominant dimension under investigation. Unidimensional biodiversity studies are neither incorrect nor inappropriate, but they are not necessarily informative about the full array of complex consequences that changes in biodiversity create. A number of recent studies demonstrate this general fact in a variety of ways and help to inform how we might better frame biodiversity research from a multidimensional perspective. Of course, multidimensional research is empirically more challenging because of the greater data demands that multivariate research invariably poses. However, with increasing improvements in biodiversity data acquisition and sharing, the rise of multi-institutional and multi-investigator research, and increasing sharing of data and open source software tools, the challenges of multidimensional biodiversity research can be readily met. With the advent of further multidimensional studies, our understanding of its importance and added value over unidimensional studies will become clearer. Given the strong dominance of unidimensional past and present biodiversity research, it is likely that we have only begun to illuminate the environmental consequences of biodiversity at the heyday of contemporary mass extinction.

## Supplementary Material

Biodiversity as a multidimensional construct: Alternative SEM Models, Placement of Number of Taxa, and Correlations

## Data accessibility

Trait data are available at https://www.try-db.org/TryWeb/Home.php.
