# Supplementary material for: Biodiversity as a multidimensional construct: a review, framework and case study of herbivory's impact on plant biodiversity
Source: Proc Biol Sci. 2016 Dec 14;283(1844):20153005. doi: 10.1098/rspb.2015.3005 (PMC5204135; doi:10.1098/rspb.2015.3005)
Supplement: Biodiversity as a multidimensional construct: Alternative SEM Models, Placement of Number of Taxa, and Correlations [file rspb20153005supp1.pdf]

Biodiversity as a multidimensional construct: Alternative SEM Models and Placement of Number of Taxa

S. Naeem, Case Prager, Brian Weeks, Alex Varga, Dan F.B. Flynn, Kevin Griffin, Robert Muscarella, Matthew Palmer, Stephen Wood, William Schuster.

In Structural Equation Modeling (SEM), each model represents a hypothetical set of causative and correlative relations (linkages) among measured and unmeasured variables. In the absence of any specific model of interest, there are several possible models, each reflecting possible alternative patterns of linkages among variables. Thus, one can examine a specific model that reflects a specific structure or one can explore all identified, admissible models to find the model that best fits or predicts the data. "Identified" means that the number of known parameters exceeds the number of unknown parameters, thus the model may be resolvable. In many cases, however, even if the number of knowns exceeds unknowns, the data may yield an inadmissible model. A model is deemed inadmissible if the analytical algorithm yielded negative residual variances, an $R^2 > 1$, or an otherwise inadmissible solution [1]. In some cases, a model is excluded because it could not be resolved because the iteration limit set by the algorithm was reached and increasing the limit did not change the outcome. Thus, many models can be constructed for a single set of variables, though generally only a fraction of them make sense (e.g., biologically), can be identified, provide admissible results for a specific set of data, and can be resolved within the iteration limit of the algorithm.

This exploratory approach in which many models are explored for a single set of variables, or exploratory SEM, can identify the best predictive model for a specific data set. The most predictive model, however, may not reflect the full set of linkages that are relevant; it only reflects linkages supported by the data. A different set of data may yield a different model as the best predictor. The full model and its rationale are therefore critical parts of the exercise (for a discussion of these and related issues concerning SEM, see [2-7]).

In this study, the SEM-framework (Fig. 2) and its rationale are explained in the main text. Our single, full model (Fig. 3, Fig. S1 A) is derived directly from the SEM framework and is based, in part, on the biological argument that the number of taxa (i.e., species richness) covaries with most, if not all biodiversity metrics (Fig S1 A and Fig. 2). However, there may be reasons to treat the number of taxa differently. The number of taxa may be treated as a variable that directly influences the ecosystem property (Fig. S1A). Alternatively, the number of taxa may be treated as a variable that directly influences biodiversity metrics (Fig S1 C). Finally, another alternative would be to treat the number of taxa as a variable that directly influences the taxonomic diversity dimension (TD), but not functional or phylogenetic diversity dimensions (FD or PD, respectively, Fig. S1 D). There are many more alternatives, but we examine these four (Fig. S1 A-D) to serve two purposes. First, we examine these alternatives to address possible differences in the way researchers may wish to treat number of taxa in multidimensional biodiversity analyses. Second, we simply wish to illustrate the exploratory approach as a supplementary exercise.

Methods

We performed an exploratory SEM approach in which we examined the four alternative models shown in Figure S1 and described above. We used the Specification Search function in the SEM statistical software package, Amos [1], to search for alternative structures for each of the four models to determine if there were alternatives that better fit our data (or were potentially more predictive) than the full model. Alternative structures consisted of the full set of possible structures in which one or more links were removed. Only models that are identified and admissible were considered. Unresolved models that occurred because the iteration limit, initially set at 50 iterations, were rerun with the limit set to 100. This procedure did not increase the number of models resolved, thus we considered these models as effectively unresolvable for the data to hand.

Results

In Table S1, we provide the total number of possible alternative models for each of the four models shown in Figure S1. There are thousands of possible alternative models for each model shown in Figure S1 (Table S1), the vast majority of which are either unidentified, inadmissible, or failed to be resolved before the algorithm's iteration limit was reached. The model treating number of taxa as a covariate (Fig. S1 A) was identified and admissible for plots with or without exposure to herbivory, but the alternatives (Fig. S1 B-D) did not provide interpretable results (Table S1). By "interpretable," we mean being able to compare model outcomes between plots exposed to or protected from herbivory. Only one full model was identified; the model presented in Figure S1 D in which number of taxa was linked to the taxonomic diversity dimension (TD) for plots protected from herbivory. Because the full model for plots exposed to herbivory was inadmissible, we cannot compare whether different dimensions had different influences on the ecosystem property (total cover) between the two treatments. Selecting admissible alternatives would allow for predicting total cover based on diversity metrics or dimensions, but our question concerned how the full models compared, not how best to predict total cover. All models, full and alternatives, rejected the hypothesis that they fit the data, thus, as discussed in the text, that although model fit methods in SEM remain under discussion [8-10], specific predictions could be suspect.

Discussion

Different researchers may have cause to deviate from our suggested generic model (Fig. 2), but for our specific example, three alternative approaches (Fig. S1 B-D) in which the number of taxa (i.e., species richness) was treated differently, did not provide interpretable results. The large number of inadmissible and unresolved models most likely stems from insufficient data and it is possible the alternative treatments of number of taxa we explored may work where more data are available.

It is important to note that we cannot, in our case study, shed light onto whether alternative models (Fig S1 B-D) given the data we have. We have provided our rationale for the model presented in Fig. 2, but further work, different systems, and different questions may lead to alternative models better suiting

different investigations. We also do not advocate nor discourage exploratory analyses of multi-dimensional models of biodiversity's influence on ecosystem function as done here in this supplementary material for our case study. We focused our analyses on testing the biologically plausible model in which the number of taxa was considered a covariate of all biodiversity metrics (Fig. 2) and the data applied to the full models showed multidimensional biodiversity effects to differ between herbivore-exposed and herbivore protected plots. However, when an alternative model is more appropriate for the hypothesis under investigation, such as any of the three alternatives illustrated in Figure S1 (B-D), such models should be examined. As in all SEM-based studies, the number of possible models can be fairly large and many may not be identifiable (too many unknowns) or admissible (unresolvable by analytical algorithms when sample sizes are small), as in our case.

Tables

Table S1. Exploratory Structural Equation Model (SEM) for multiple dimensions of biodiversity applied to understory forest vegetation at Black Rock Forest, New York, exposed to or protected from deer herbivory. For each SEM model in which the treatment of number taxa is different (Fig. S1 A-D), a specification search was conducted using Amos and basic outcomes of the search are tabulated here. AIC is the is the Aikake Information Criterion which measures the likelihood of the model given the data and aids in model selection among a set of comparable models The AIC for the best model is provided (maximum AIC is provided parenthetically); lower values reflect better fits. Herbivory was either present (i.e. "Yes") or not (i.e., exclosures were present, "No"). P reflects probability that model fits the data if P (<0.05 means that model was rejected as a good fit to the data). Results for the full model (i.e., all links illustrated in Fig. S1 A-D are present) are provided in the right most column. If the full model was admissible, the R^2 for the model's prediction of total cover is provided parenthetically.

				Best Fit Model			Full model
	**Number of models**	**Number of Admissible Models**	**Her-bivory**	**Para-meters**	**AIC of best fit (poorest fit) model**	**P**	**Admissible (R^2) or Inadmissible**
Covariate with Dimensions	32,768	14	Yes	21	174.0 (187.1)	<0.001	Admissible (0.76)
		7	No	16	179.0 (187.1)	<0.001	Admissible(0.19)
Directly influences Total Cover (Fig. S1 B)	8,192	8	Yes	17	167.6 (176.5)	<0.001	Inadmissible
		7	No	20	170.2 (187.1)	<0.001	Inadmissible
Influences Dimensions (Fig. S1 C)	262,144	163	Yes	18	117.9 (176.5)	<0.001	Inadmissible
		112	No	21	127.4 (179.0)	<0.001	Inadmissible
Directly influences TD (Fig. S1 D)	8,192	6	Yes	14	169.4 (177.4)	<0.001	Inadmissible
		3	No	16	179.0 (187.1)	<0.001	Admissible (0.65)

Table S2 Correlation matrix for biodiversity metrics used in structural equation modeling example (see Fig. 3). Pearson correlations. Bolded numbers mean $P < 0.05$, Bonferroni corrected.

	**Shannon**	**Simpson**	**FD Divergence**	**FD Evenness**	**FD Richness**	**Phylogenetic Faith**	**Phylogenetic MPD**
Simpson	**0.96**						
FD Divergence	-0.01	-0.14					
FD Evenness	0.07	0.02	-0.10				
FD Richness	0.48	0.33	0.58	-0.14			
Phylogenetic Faith	0.49	0.33	0.58	-0.14	**0.97**		
Phylogenetic MPD	**0.93**	**0.88**	0.07	0.01	0.60	**0.62**	
Number of Taxa	0.52	0.37	0.53	-0.18	**0.95**	**0.97**	0.59

Figure Captions

Figure S1. Four possible SEM models for the relationship between multiple dimensions of biodiversity and an ecosystem property; TD, FD, and PD related to total vegetation cover in understory vegetation plots at Black Rock Forest. See caption to Figure 3 for further detail. A. Number of species is a covariate of TD, FD, and PD. B. Number of taxa as a variable that directly influences the ecosystem property. C. Number of taxa directly influences biodiversity metrics. D. Number of taxa directly influences the taxonomic diversity dimension.

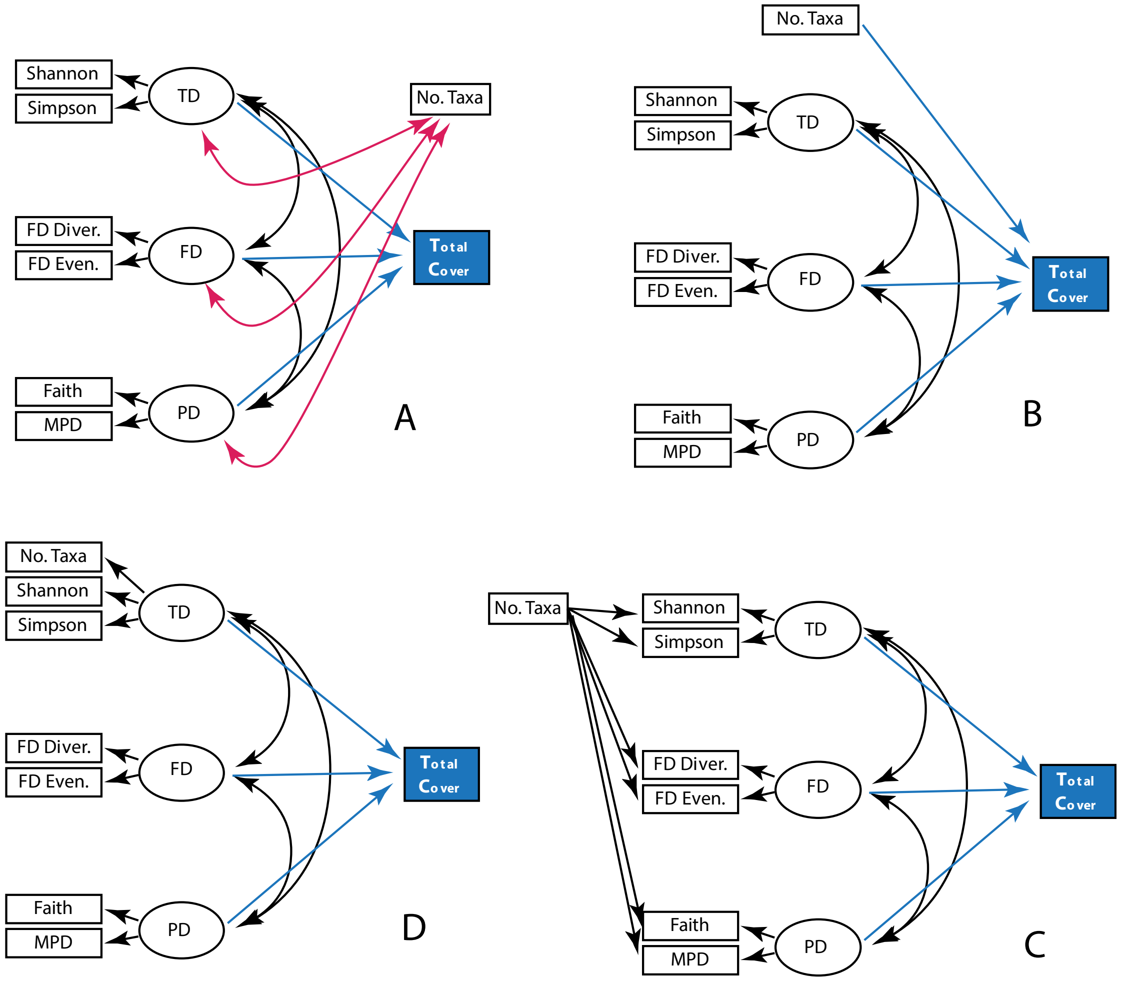
Shannon
Simpson
TD
FD Diver.
FD Even.
FD
Faith
MPD
PD
No. Taxa
Total Cover
A
No. Taxa
Shannon
Simpson
TD
FD Diver.
FD Even.
FD
Faith
MPD
PD
Total Cover
B
No. Taxa
Shannon
Simpson
TD
FD Diver.
FD Even.
FD
Faith
MPD
PD
Total Cover
D
No. Taxa
Shannon
Simpson
TD
FD Diver.
FD Even.
FD
Faith
MPD
PD
Total Cover
C

Figure S1

Literature cited

1. Arbuckle J.L., Wothke W. 1999 *Amos 4.0 Users Guide*. Chicago, SmallWaters Corporation; 452 p.
2. Grace J.B., Schoolmaster D.R., Guntenspergen G.R., Little A.M., Mitchell B.R., Miller K.M., Schweiger E.W. 2012 Guidelines for a graph-theoretic implementation of structural equation modeling. *Ecosphere* **3**(8), art73. (doi:10.1890/ES12-00048.1).
3. Byrne B.M. 2013 *Structural equation modeling with AMOS: Basic concepts, applications, and programming*, Routledge.
4. Marcoulides G.A., Schumacker R.E. 2013 *Advanced structural equation modeling: Issues and techniques*, Psychology Press.
5. Grace J.B., Pugseek B.H. 1997 A structural equation model of plant species richness and its application to a coastal wetland. *American Naturalist* **149**, 436-460.
6. Shipley B. 2001 *Cause and Correlation in Biology: A User's Guide to Path Analysis, Structural Equations, and Causal Inference.* Cambridge, Cambridge University Press; 316 p.
7. Hershberger S.L., Marcoulides G.A., Parramore M.M. 2003 Structural equation modeling: An introduction. In *Structural Equation Modeling* (eds. Pugesek B.H., Tomer A., EVon Eye A.). Cambridge, Cambridge University Press.
8. Barrett P. 2007 Structural equation modelling: Adjudging model fit. *Personality and Individual Differences* **42**(5), 815-824. (doi:http://dx.doi.org/10.1016/j.paid.2006.09.018).
9. Moshagen M. 2012 The Model Size Effect in SEM: Inflated Goodness-of-Fit Statistics Are Due to the Size of the Covariance Matrix. *Structural Equation Modeling: A Multidisciplinary Journal* **19**(1), 86-98. (doi:10.1080/10705511.2012.634724).
10. Kenny D.A., Kaniskan B., McCoach D.B. 2015 The Performance of RMSEA in Models With Small Degrees of Freedom. *Sociological Methods & Research* **44**(3), 486-507. (doi:10.1177/0049124114543236).
